# Effectiveness and tolerability of radiotherapy for patients with indolent non-Hodgkin’s lymphoma: a monocenter analysis

**DOI:** 10.1038/s41598-021-01851-w

**Published:** 2021-11-19

**Authors:** I. Hadi, A. Schummer, M. Dreyling, C. Eze, R. Bodensohn, O. Roengvoraphoj, C. Belka, M. Li

**Affiliations:** 1 Department of Radiation Oncology, University Hospital, LMU Munich, 81377 Munich, Germany; 2 Department of Internal Medicine III - Oncology, University Hospital, LMU Munich, Munich, Germany; 3 Radiation Oncology, Dachau, Germany; 4 German Cancer Consortium DKTK, Munich, Germany

**Keywords:** Non-hodgkin lymphoma, Non-hodgkin lymphoma

## Abstract

To analyze the effectiveness and toxicities of radiotherapy in indolent non-Hodgkin’s lymphoma (iNHL) patients treated in our institution. Patients with iNHL treated with radiotherapy between 1999 and 2016 were included. The primary endpoint was progression-free survival (PFS). Secondary endpoints were local control (LC), overall survival (OS) and toxicities. PFS, LC, and OS were analyzed using Kaplan–Meier method. Log-rank test was used to investigate the differences between subgroups. Cox proportional hazard model was used for univariate continuous analysis. Seventy-five patients were identified in our institutional database between 1999 and 2016. Fifty-eight (77.3%) had stage I after Ann-Arbor and 17 patients (22.7%) had stage II. The median follow-up was 87 months (95% CI 72–102 months). Median single dose per fraction was 2.0 Gy (range 1.5–2 Gy) and median total dose was 30.6 Gy (range 16–45 Gy). Radiotherapy was performed in 2D (n = 10; 13.3%), 3D (n = 63; 84.0%) and VMAT (n = 2; 2.7%) techniques, respectively. The median PFS was 14.0 years (95% CI 8.3–19.7 years). The estimated PFS after 5 and 10 years were 73.0% and 65.5% in Kaplan–Meier analysis, respectively. The 5- and 10-year LC were 94.9% and 92.3%, respectively. The 5- and 10-year OS were 88.6% and 73.9%. In univariate analyses of PFS, younger patients (≤ 60 years old) had significantly superior PFS to those older than 60 years old (5-year PFS 81.9% vs. 65.1%, p = 0.021). Dose escalation > 36.0 Gy had no prognostic influence in term of PFS (p = 0.425). Extranodal involvement, stage and histology had no prognostic impact on PFS. Depending on the site of lymphomas, the most common acute side effects were: dermatitis CTCAE° I–II (8.0%), xerostomia CTC° I (8.0%), cataract CTC° I (12.0%) and dry eyes CTC° I–II (14.6%). No adverse event CTC° III was reported. Most acute side effects recovered at 3 to 6 months after radiotherapy except for CTC° I cataract and xerostomia. Local Radiotherapy was highly effective for treatment of early stage iNHL with no serious side effects in our cohort. The most acute CTCAE° I–II side effects recovered 3 to 6 months later. Technique advances seem to have further improved effectiveness and tolerability of radiotherapy.

**Trial registration:** Local ethics committee of Ludwig-Maximilian-University (LMU) Munich approved this retrospective analysis on the May 7th, 2019 (Nr. 19–137).

## Introduction

Indolent non-Hodgkin’s lymphoma (iNHL) is a heterogeneous group of diseases arising from lymphoid tissue, which is characterized by prolonged survival over years or decades^1^. Follicular lymphoma (FL) and extranodal MALT lymphoma are the most common histologies^1^. Of all FL, localized stage (stage I–II) was found in approximately 15–20%^1^. Both FL and MALT were considered as radiosensitive neoplasia, so that a relative low dose radiotherapy achieves excellent local control, applied either as a curative approach in early stage or as a palliative measure in advanced stage^2,3^. NCCN as well as ESMO guidelines recommend Radiotherapy (RT) as the first choice of curative-intended treatment for iNHL in early stages^4–7^. However, a retrospective cohort study of National Cancer Data Base reported a decline in the use of RT in patients with early stage FL from 37 in 1999 to 24% in 2012^8^. In order to show the effectiveness and side effects of RT, we retrospectively analyzed the clinical outcomes of iNHL patients, treated with RT in our department during the last two decades.

## Patients and methods

### Patients

Patients with non-Hodgkin lymphoma, who underwent radiotherapy between 1999 and 2016, were identified from the institutional database. We excluded patients with aggressive lymphoma, iNHL stage III and IV, as well as follow up less than 3 months. Patient demographics, tumor characteristic, and comprehensive treatment parameters were collected for analysis. Informed consent was obtained from all patients and local ethics committee of Ludwig-Maximilian-University (LMU) Munich approved this retrospective analysis on the May 7th, 2019 (Nr. 19–137).

### Statistical analysis

Patient demographics were calculated using descriptive statistics as absolute and relative frequencies. The primary endpoint of this study was progression-free survival (PFS). PFS was a time-to-event endpoint and defined as the interval between the beginning of radiotherapy to the earliest date of progressive disease, relapse, or death resulting from any cause^9^. PFS, LC, and OS were analyzed using Kaplan–Meier method. Log-rank test was performed to investigate the differences between subgroups. Chi-square and Cramer’s V were utilized to analyze association of nominal parameters. Cox proportional hazard model was used for univariate continuous analysis. A two tailed p-value of < 0.05 was considered significant. We performed statistical analyses with IBM SPSS Statistics, Version 25 (IBM, Armonk, New York, USA). All methods were carried out in accordance with relevant guidelines and regulations.

### Ethics approval

Local ethics committee approved this retrospective analysis on 7th of May 2019 (Nr. 19–137).

## Results

### Patient characteristics

In the initial database screening, 574 lymphoma patients treated with radiotherapy in our department between 1999 and 2016 were identified. After exclusion of aggressive lymphoma, patients with stage III and IV, and follow up less than 3 months, 75 patients with stage I or II iNHL remained for retrospective analysis. Median follow up was 87 months (95% CI 72–102 months). A CONSORT (Consolidated Standards of Reporting Trials) diagram of our cohort is presented in Suppl. Fig. 1.

Median age by the first diagnosis was 61 years (range 24–92 years). Twenty-eight patients were male (37.3%) and 47 patients were female (62.7%). Most of the patients (n = 74, 98.7%) had a good performance status (ECOG 0–1) while1 patient (1.3%) had ECOG 2. Regarding the histology, follicular lymphoma (FL) was found in 45 patients (60.0%) and marginal zone lymphoma (MZL) in 30 patients (40.0%). We conducted Chi-square to test differences between FL and MZL at the baseline. We found more extranodal involvement in patients with MZL (n = 27) than in patients with FL (n = 13), p ≤ 0.001. Following extranodal sites were observed: orbita, stomach, vallecula epiglottica, parotid and submandibular gland, intrapulmonary, prevertebral, bone, and upper arm lesion.

In order to rule out systemic involvement, PET/CT were performed in 19 patients (25.3%) and 56 patients (74.7%) underwent contrast enhanced whole body computer tomography (CT). We observed an increasing number of PET/CT utilization over the years. Only 5 from 36 patients (14%), who were treated before 2010, underwent PET/CT at the diagnosis. In comparison, PET/CTs were performed in 14 from 39 patients (36%), who were treated in 2010 and afterwards.

Fifty-eight patients (77.3%) harbored stage I Ann Arbor lymphoma, and 17 patients (22.7%) stage II. B-symptoms were reported in 3 patients (4.0%) and extranodal involvement in 40 patients (53.3%). Bulky disease was defined as largest lymph node > 6 cm^10^. We observed 7 patients (9.3%) patients with bulky disease in our cohort. Four patients had elevated LDH level (5.3%), 49 patients (65.3%) had normal LDH value and 22 patients (29.3%) had no documented LDH value.

We applied Follicular Lymphoma International Prognostic Index (FLIPI) for patients with FL and observed 34 patients (75.6%) with low-risk, 6 patients (13.3%) with intermediate-risk, and 5 patients (11.1%) with unknown FLIPI score due to unknown LDH. We used Marginal Zone Lymphoma of Mucosa-associated Lymphoid Tissue International Prognostic Index (MALT-IPI) for MZL, and it resulted in 15 patients (50.0%) with low-risk, 11 patients (36.7%) with intermediate-risk and 4 patients (13.3%) with unknown risk (due to unknown LDH).

In 58 patients (77.3%), lymphoma manifestation was found in one lymph node region, 11 patients (14.7%) had lymphoma in 2 LN region, 6 patients (8.0%) had lymphoma in ≥ 3LN region.

Patients’ characteristics are summarized in Table 1.

**Table 1:** Patients’ characteristics.

Characteristic	Number of patients (n = 75)	
	Absolute (n)	Relative (%)
**Sex**		
Male	28	37.3
Female	47	62.7
**ECOG**		
0–1	74	98.7
2	1	1.3
**Histology**		
Follicular	45	60.0
Marginal zone	30	40.0
**Staging**		
Whole body CT	56	74.7
PET/CT	19	25.3
**Ann Arbor staging**		
I	58	77.3
II	17	22.7
**B-type symptoms**		
Yes	3	4.0
No	72	96.0
**Extranodal**		
Yes	40	53.3
No	35	46.7
**Elevated LDH**		
Yes	4	5.3
No	49	65.3
Unknown	22	29.3
Bulky disease	7	9.3%
**FLIPI**		
Low risk	34	75.6
Low-intermediate risk	6	13.3
Unknown	5	11.1
**MALT-IPI**		
Low risk	15	50.0
Low-intermediate risk	11	36.7
Unknown	4	13.3
**Number of lymphoma manifestation**		
1 Lymph node region	58	77.3
2 LN region	11	14.7
≥ 3 LN region	6	8.0

*FLIPI* Follicular Lymphoma International Prognostic Index, *MALT-IPI* Marginal Zone Lymphoma International Prognostic Index.

### Treatment parameters

Sixty-six patients (88.0%) received radiotherapy as primary treatment and 9 (12.0%) patients underwent irradiation in recurrent situation.

Among 66 patients, who underwent RT in primary situation, 49 patients (74.2%) were treated with RT only. Eight patients (12.1%) underwent resection prior to RT, 2 (3.0%) patients received systemic therapy prior to RT (rituximab or R-CHOP), 4 patients (6%) received concurrent rituximab to RT. Systemic therapy with bendamustin/rituximab was given sequentially post RT in 1 patient (1.5%). Two patients (3.0%) underwent resection and systemic therapy prior to RT.

In 9 patients who received RT in recurrent situation, 6 patients (66.7%) were treated with systemic therapy prior to RT and 3 patients (33.3%) underwent resection prior to RT.

Radiotherapy was performed with a median single dose of 2 Gy (range 1.5–2 Gy) and a median total dose of 30.6 Gy (range 16–45 Gy). Target volume delineation was based on involved-field radiation therapy (IFRT) in 43 patients (57.3%) and involved-site radiation therapy (ISRT) in 32 patients (42.7%). Radiotherapy planning was simulator-based (2D-RT) in 10 cases (13.3%), three dimensional (3D-RT) in 63 cases (84.0%), and volumetric modulated arc therapy (VMAT) in 2 cases (2.7%). Radiation with 6 MeV beam was performed in 48 cases (64.0%) while in other 27 cases with > 6 MeV beam (36.0%).

As for radiation volume, 40 patients (53.3%) underwent radiation in extranodal regions, 18 patients (24.0%) in inguinal or femoral lymph node regions, and 10 patients (13.3%) in cervical and supraclavicular lymph node regions. Radiation of other lymph node regions, such as Waldeyer’s ring, axillary, paraaortic, mesenteric and iliac lymph node region was applied in 7 patients (9.3%). All patients with stage II disease received RT in one target volume. The summary of treatment parameters are described in Table 2.

**Table 2:** Treatment parameters.

Parameters	Number of patients (n = 75)	
	Absolute (n)	Relative (%)
**Irradiation in primary vs recurrent treatment**		
RT as primary treatment	66	88.0
RT only	49	74.2
Resection prior to RT	8	12.1
Systemic therapy prior to RT	2	3.0
Rituximab	(1)	
R-CHOP	(1)	
Concurrent Rituximab to RT	4	6.0
Systemic therapy sequentially given post RT	1	1.5
Bendamustin/rituximab		
Resection and systemic therapy prior to RT	2	3.0
Resection → Rituximab	(1)	
Resection → R-CVP	(1)	
RT as recurrent treatment	9	12.0
Systemic therapy prior to RT	6	66.7
Resection prior to RT	3	33.3
**Single dose per fraction (Gy)**		
1.5	4	5.3
1.8	33	44.0
2.0	38	50.7
Median (range)	2.0 Gy (1.5–2.0 Gy)	
**Total dose of radiation therapy (Gy)**		
< 24 Gy	1	1.3
24–36.0 Gy	46	61.3
> 36.0 Gy	28	37.3
Median (range)	30.6 Gy (16–45 Gy)	
**Technique of irradiation**		
2D—RT	10	13.3
3D—RT	63	84.0
VMAT	2	2.7
**Energy beam**		
6 MeV	48	64.0
> 6 MeV	27	36.0
**Radiation field**		
IFRT	43	57.3
ISRT	32	42.7
**Target volume**		
Extra nodal regions	40	53.3
Inguinal or femoral lymph node region	18	24.0
Cervical of supraclavicular LN region	10	13.3
Other LN region	7	9.3

### Progression-free survival

**Figure 1:**
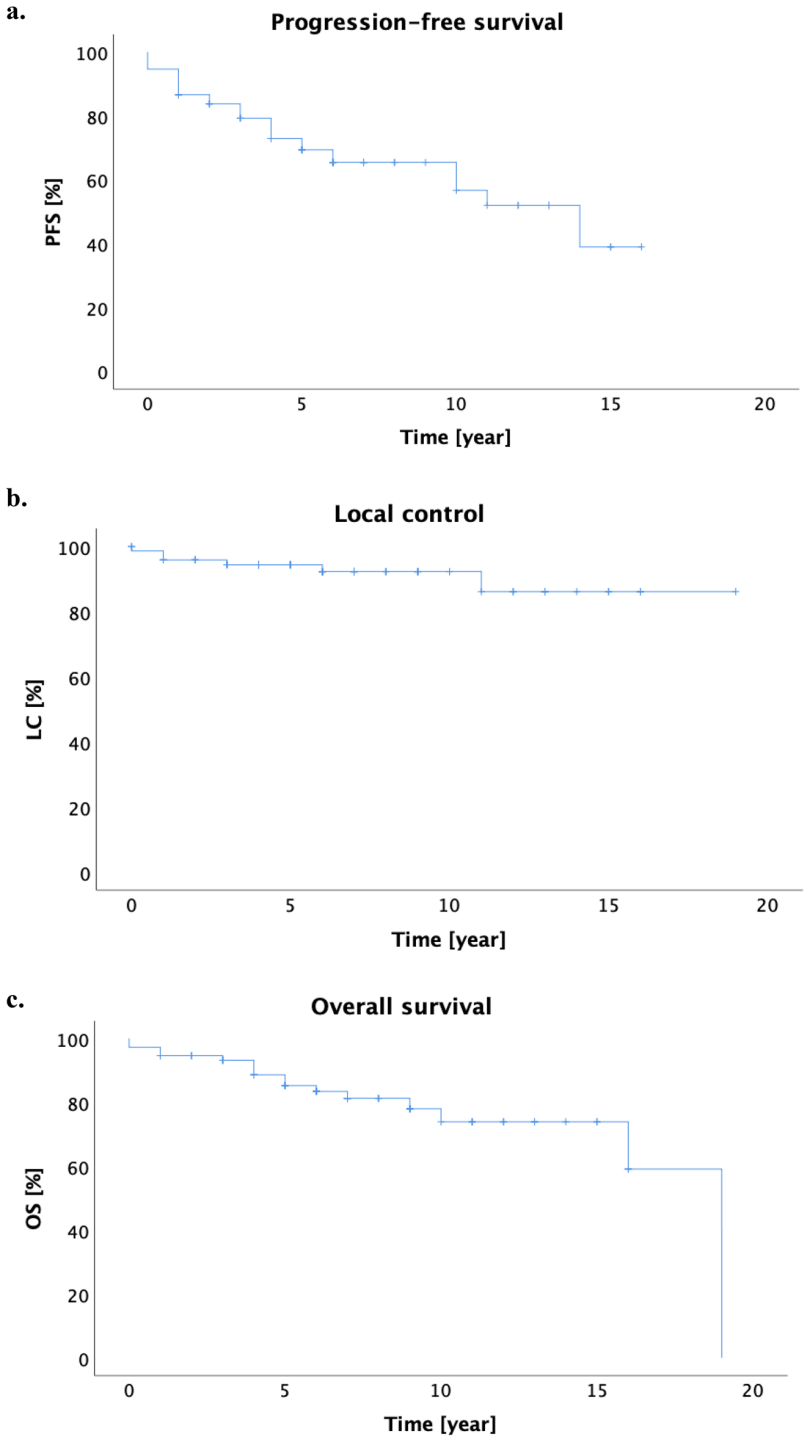
(**a**) The estimated Kaplan–Meier PFS for 5 and 10 years were 73.0% and 65.5% respectively. (**b**) The 5- and 10-year LC was 94.4% and 92.3% respectively. (**c**) The 5- and 10-year OS were 88.6% and 73.9%.

**Figure 2:**
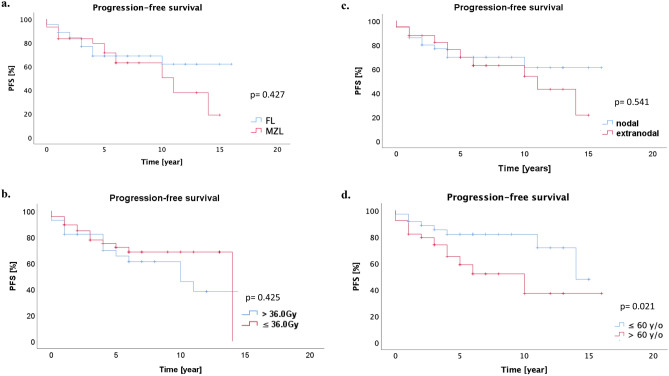
(**a**) Different lymphoma subtypes achieved a comparable long-term outcome (5-year PFS for FL 68.8% vs. MZL 79.4%, p = 0.427). (**b**) Total radiation dose of > 36.0 Gy had no prognostic influence to PFS (5-year PFS 65.5% vs. 72.1% for > 36.0 Gy vs ≤ 36.0 Gy, p = 0.425). (**c**) Extranodal involvement was not associated with inferior PFS (5-year PFS 76.0% vs. 69.6% for extranodal and nodal involvement, p = 0.541). (**d**) Patients who were younger than or equal to 60 y/o had significantly improved PFS compared to patients who were older than 60 y/o (5-year PFS 81.9% and 65.1%, p = 0.021).

The Kaplan–Meier estimates of 5- and 10-years PFS were 73.0% and 65.5%, respectively. The median PFS was 14 years (95% CI 8.3–19.7 years, Fig. 1a). In 49 patients, who underwent RT alone, the 5- and 10-years PFS were 81.0% and 67.4%. Different lymphoma subtypes achieved a comparable long-term outcome (5-year PFS for FL 68.8% vs. MZL 79.4%, p = 0.427, Fig. 2a). Dose escalation > 36.0 Gy had no prognostic influence to of PFS than ≤ 36.0 Gy (5-year PFS 65.5% vs. 72.1%, p = 0.425, Fig. 2b). There was no significant difference between patients with nodal and extranodal iNHL in term of PFS (5-year PFS 69.6% vs. 76.0% for nodal and extranodal iNHL, p = 0.541, Fig. 2c). Younger patients (≤ 60 years old) had significantly superior PFS to those older than 60 years old (5-year PFS 81.9% vs. 65.1%, p = 0.021, Fig. 2d). ISRT was not inferior to IFRT (p = 0.543). Univariate analysis of PFS was summarized in Table 3.

**Table 3:** Univariate analysis of PFS.

PFS			
Parameters	Univariate analysis		
	HR (95%CI)	Median PFS in years (95% CI)	p-value
**Gender**			
♂	1.3 (0.6–2.9)	10 (3.6–16.3)	0.432
♀		14 (2.8–25.2)	
**Age**			
≤ 60 y/o	2.5 (1.1–5.8)	14.0 (10.2–14.8)	0.021
> 60 y/o vs		10.0 (5.1–14.8)	
**Histology**			
FL	1.3 (0.6–2.8)	Not reached	0.427
MZL		11.0 (5.2–16.8)	
**Ann Arbor staging**			
St. I	0.9 (0.6–1.5)	11.0 (7.4–14.6)	0.751
St. II		Not reached	
**Extranodal**			
Yes	1.2 (0.6–2.7)	11.0 (4.5–17.5)	0.541
No		Not reached	
**RT as primary vs recurrent**			
Primary treatment	1.9 (0.7–5.1)	14.0 (8.3–19.7)	0.179
Recurrent treatment		10.0 (3.8–10.0)	
**Total dose of RT**			
≤ 36.0 Gy	1.4 (0.6–2.9)	14.0 (8.7–19.7)	0.425
> 36.0 Gy		10.0 (4.9–15.1)	
**Technique of RT**			
3D/VMAT	0.3 (0.1–0.8)	14.0 (8.2–19.8)	0.059
2D		4.0 (1.1–6.9)	
**Radiation field**			
IFRT	1.3 (0.6–2.7)	Not reached	0.543
ISRT		10 (4.3–15.7)	

We performed subgroup analyses with patients who received RT at the frontline, and precluded four patients, who received systemic therapy prior to RT (rituximab or R-CHOP), or underwent resection or systemic therapy prior to RT. In this subgroup of 62 patients, 5- and 10-years PFS were 76.1% and 64.5%, respectively. The subgroup analyses also demonstrated, that patients who received dose escalation of > 36 Gy had no better PFS than ≤ 36 Gy (p = 0.466). In the further univariate analyses of PFS, we did not find significant prognostic role of histology (FL vs. MZL), age (> 60 years old vs. younger) and nodal vs. extranodal. ISRT was not inferior to IFRT. In 9 patients, who received RT in the recurrent setting, 5- and 10-years PFS were 55.6% and 0% respectively.

### Local control and overall survival

Local progress after radiotherapy was reported in 6 patients (8.0%). The 5- and 10-year LC was 94.4% and 92.3%, respectively, the median was not reached (Fig. 1b). Bases on histologies, 5- and 10-years local control were 95.2% and 95.2% for FL, versus 93.2% and 88.6% for MZL (p = 0.187), respectively. In 62 patients, who received RT at the frontline, local control after 5- and 10-years were 96.7% and 96.7%. The 5- and 10-years LC in patients, who received RT in the recurrent setting (n = 9), were 88.9% after 5 and 10 years. In patients who underwent RT alone (n = 49), LC after 5 and 10 years were 97.9% and 97.9% respectively.

Outfield progress was described in 15 patients (20.0%). Among these patients, 10 patients had FL (stage I Ann Arbor = 7 patients; stage II = 3 patients), and 5 patients had MZL (stage I Ann Arbor = 4 patients; stage II = 1 patient). Based on the FLIPI, 5 patients were in the low risk group, 4 patients in the intermediate risk, and 1 patient had unknown FLIPI. In term of MALT-IPI, 4 patients were in the low risk group and 1 patient had unknown MALT-IPI. Two of 15 patients with distant recurrent had bulky disease. Four patients received systemic therapy with R-CHOP prior to radiation. Extranodal involvement was found in 7 patients.

Sixteen patients (21.3%) were deceased at the time of last follow up. The 5- and 10-year OS were 88.6% and 73.9% respectively, with a median of 19 years (Fig. 1c). Based on different histologies, the 5- and 10-years OS were 90.2% and 77.2% for FL, versus 86.3% and 70.3% for MZL (p = 0.156), respectively. The 5- and 10-years OS in 62 patients, who received RT at the frontline, were 91.6% and 74.5% respectively. In patients, who underwent RT at the recurrent, the 5- and 10-years OS were 63.5% and 0% respectively.

### Toxicity

Depending on the site of lymphoma, the most common acute side effects were: dermatitis CTCAE° I–II (n = 6; 8.0%), xerostomia CTC° I (n = 6; 8.0%), cataract CTC° I (n = 9; 12.0%), and dry eyes CTC° I–II (n = 11; 14.6%). No adverse event CTC° III was reported. Most of acute side effects recovered at 3 to 6 months after radiotherapy except for CTC° I cataract, dermatitis, and xerostomia. The summary of toxicities after radiotherapy is described in Table 4.

**Table 4:** Acute side effects of radiation therapy according to Common Terminology Criteria for Adverse Events (*CTCAE*) v4.0.

Acute toxicity	CTCAE°		Remaining toxicity at 3–6 months after RT
	I	II	
Dermatitis	5.3% (4)	2.7% (2)	1.3% (1)
Dysphagia	5.3% (4)		
Xerostomia	8.0% (6)		5.3% (4)
Lymphedema	2.7% (2)		
Pneumonitis	1.3% (1)		
Dysuria	1.3% (1)		
Cataract	12.0% (9)		12.0% (9)
Dry eyes	13.3% (10)	1.3% (1)	
Alopecia	1.3% (1)		

Using Chi-square and Cramer’s V statistical methods, we analyzed the correlation between toxicities, radiation dose and extranodal involvement. We did not find any significant difference regarding toxicity between > 36.0 and ≤ 36.0 Gy (p = 0.197). There were significant more side effects for extranodal involvements (CTCAE° I–II: 57.5% in extranodal cohort vs 28.6%, p = 0.012).

## Discussion

In the present study, we report that radiotherapy provided excellent LC, PFS and OS for iNHL, with no difference between FL and MZL, as the two major subgroups. These results were in accordance with those from several preceding studies^11–20^. However, PFS decreased from 73.0 at 5 years to 65.5% at 10 years. Similar results were also reported by some previously published studies^11–18^. The discrepancy between LC and PFS was mainly caused by distant progression outside of radiation volume, which was observed in 15 patients (20.0%) in our cohort with a median time of 87 months after RT.

The issue of distant relapses after radiotherapy has raised the question whether adding systemic therapy might help to improve PFS. A German phase II MIR trial combined anti-CD20 antibody rituximab with involved-field radiotherapy (IFRT, 30–40 Gy)^21^. IFRT combined with rituximab was well tolerated and 5-years PFS was 78%, which was slightly higher than our results^22^. The potential improvement of PFS by adding rituximab to IFRT (30–40 Gy) was also confirmed by Ruella et al.^23^. However, this observational multicenter study showed no OS difference between RT alone and RT combined with rituximab. A more recent randomized trial, comparing IFRT alone with IFRT plus immunochemotherapy (rituximab, cyclophosphamide, vincristine, and prednisolone), reported significantly better 10-years PFS in the arm of combined therapy (59% vs 41%), albeit at cost of grade III or IV acute toxicities (65% in the combined arm)^24^. In contrast, we did not observe any grade III or higher toxicities in the present study, in which the majority was treated with RT alone.

The advance in diagnostic imaging enabled better localization of involved lymph nodes and radiation volumes. Supported by the development of more sophisticated RT technique (e.g. 3D, IMRT, VMAT, instead of 2D) it was possible to further reduce RT volumes from IFRT to ISRT. In our cohort, target volume delineation were performed according to IFRT (57.3%) and ISRT (42.7%) strategies respectively. In our univariate analysis, ISRT was not inferior to IFRT in term of PFS. This stands in accordance with a retrospective analysis of more than 200 patients, which showed the non-inferiority of ISRT and the most common pattern of failure in IF- and ISRT groups was distant recurrence^25^. In the current study, extranodal involvement was not associated with inferior PFS, this stands in line with a recent analysis from König et al.^26^.

Our analysis showed that radiation dose higher than 36.0 Gy did not result in any benefit of clinical outcomes. This finding was in line with a randomized phase III trial comparing 40–45 Gy with 24 Gy, which showed no difference in overall response rate, LC, PFS, or OS^27^. Another low dose radiation therapy (LDRT) study investigated further dose de-escalation and compared 24 Gy with 4 Gy in patients with indolent lymphoma^28^. However, the group of 4 Gy was inferior to 24 Gy in term of PFS. Thus, the authors concluded that 24 Gy should be considered as standard dose for definitive radiotherapy of iNHL and LDRT of 4 Gy remained an useful alternative, especially for palliative care^28,29^. However, in the era with innovation of targeted therapy and more sensitive functional PET imaging, further reduction of radiation dose is still a striving issue with the purpose of saving radiation-induced side effects. A prospective study (GAZAI) is now ongoing to examine the effectiveness of combined low-dose radiotherapy with CD20-antibody Obinutuzumab for stage I/II follicular lymphoma. For non-responder, evaluated in FDG-PET, a second radiotherapy with 36 Gy was applied for salvage treatment. The results are eagerly awaited which may help further optimizing treatment for early stages iNHL.

Because of its indolent nature, “watch and wait” strategy has also been performed in patients with early stage iNHL (Ann Arbor I and II). A retrospective analysis of 41 selected patients showed estimated survival at 5 and 10 years of 97% and 85% after deferred therapy^30^. Therapy was not initiated mostly because of physician choice, large radiation field, advanced age, and concern about toxicity^30^. Another large retrospective cohort study of National Cancer Data Base with almost 36,000 patients reported a decline in the use of RT in patients with early stage follicular lymphoma from 37 in 1999 to 24% in 2012. In contrast to the Stanford series, this study showed an improved OS after RT^8^.

Regarding toxicity, we could show in the present study that RT of indolent lymphoma was well tolerated with no serious adverse events (≥ CTCAE° III). Extranodal involvement was associated with significant increased acute toxicities; this was attributable to the fact that the majority of extranodal involvements in our cohort were orbita lymphoma and conjunctivas, in which conjunctiva as a relative radiation sensible organ nearby often-received relevant radiation dose and developed conjunctivitis. The most recent multicenter retrospective study from Brady et al. analyzed definitive radiotherapy for localized follicular lymphoma and presented similar results as the present study^31^. There were no significant adverse effects after the relative low doses as well as limited radiation fields^31^.

With its excellent effectiveness and low toxicities, our results support the pivotal role of RT in curative treatment of early stage iNHL, being in line with the recommendation in international guidelines and the results of other studies^5,6,12,32^. Despite the long follow-up of our study allowing reliable analysis of survival rates, major limitations of our study remain its retrospective nature and the limited number of patients.

## Conclusion

Local Radiotherapy was highly effective for treatment of early stage iNHL with no serious side effects in our cohort. The most acute CTCAE° I–II side effects recovered 3 to 6 months later. Technique advances seem to have further improved effectiveness and tolerability of radiotherapy.

## Supplementary Information

Supplementary Figure 1.

## Data Availability

The datasets used and analyzed during the current study are available from the corresponding author on reasonable request.
