# Supplementary material for: Effectiveness and tolerability of radiotherapy for patients with indolent non-Hodgkin’s lymphoma: a monocenter analysis
Source: Sci Rep. 2021 Nov 19;11:22586. doi: 10.1038/s41598-021-01851-w (PMC8604980; doi:10.1038/s41598-021-01851-w)
Supplement: Supplementary file 1 — Supplementary Figure 1. [file 41598_2021_1851_MOESM1_ESM.docx]

**Fig. suppl 1** CONSORT (Consolidated Standards of Reporting Trials) diagram of patients with lymphoma, treated at our department between 1999 – 2016
